# Negative school gossip, self-esteem, and exercise intention among Chinese adolescents: a mediation model

**DOI:** 10.3389/fpsyg.2026.1923752

**Published:** 2026-09-29

**Authors:** Jiaxin Ma, Xingtong Chen

**Affiliations:** 1 School of Humanities and Arts, Deyang College of Urban Rail Transit, Deyang, Sichuan, China; 2 School of Sports Training, Chengdu Sport University, Chengdu, Sichuan, China

**Keywords:** Chinese adolescents, exercise intention, mediation, negative school gossip, self-esteem, structural equation modeling

## Abstract

**Purpose:**

This study examined the associations among negative school gossip, self-esteem, and general exercise intention among Chinese adolescents, with particular attention to the mediating role of self-esteem and the relevance of these associations to physical education (PE) and school-based movement contexts.

**Methods:**

A cross-sectional online questionnaire survey was conducted among 1,713 junior middle school students in China. Negative school gossip, self-esteem, and exercise intention were assessed using established self-report scales. Data were analyzed using latent-variable structural equation modeling in Mplus 8.3. Common method bias, reliability, convergent validity, discriminant validity, and model fit were examined before testing the mediation model. Bias-corrected bootstrap analysis with 2,000 bootstrap samples was used to test the indirect association. A sensitivity analysis was conducted by controlling for gender, grade, region, parental education, only-child status, and family economic status.

**Results:**

The three-factor measurement model showed better fit than alternative measurement models, and the measures demonstrated acceptable reliability and validity. In the main structural model, negative school gossip was negatively associated with self-esteem (*β* = −0.157, *p* < 0.001) and exercise intention (*β* = −0.063, *p* = 0.031), while self-esteem was positively associated with exercise intention (*β* = 0.240, *p* < 0.001). The standardized indirect association from negative school gossip to exercise intention through self-esteem was significant [*β* = −0.038, 95% bootstrap CI (−0.055, −0.024)], accounting for 37.62% of the total association. The pattern remained stable after controlling for demographic covariates.

**Conclusion:**

Negative school gossip was associated with weaker general exercise intention among Chinese adolescents, and self-esteem partly accounted for this association. Although exercise intention was not measured specifically for PE lessons, the findings are relevant to PE because school movement contexts are highly visible and peer-evaluative settings in which reputation-based exclusion and stigma may reduce psychological safety. The results therefore suggest that supportive peer climates and protection from gossip-related exclusion may be important for inclusive PE and equitable participation in school-based physical activity.

## 1 Introduction

### 1.1 Exercise intention as a developmental and school health issue among Chinese adolescents

Adolescence is a sensitive developmental period during which health-related beliefs, peer interaction patterns, and behavioral tendencies begin to consolidate into relatively stable lifestyles. Regular physical activity is widely recognized as beneficial for adolescents’ physical fitness, cardiometabolic health, cognitive functioning, and mental well-being (Lubans et al., 2016; World Health Organization, 2020). The World Health Organization recommends that children and adolescents accumulate an average of at least 60 min of moderate-to-vigorous physical activity per day (World Health Organization, 2020). Nevertheless, insufficient physical activity remains common among adolescents worldwide. Based on pooled data from 298 population-based surveys across 146 countries and territories, Guthold et al. (2020) reported that more than four fifths of school-going adolescents aged 11–17 years did not meet current physical activity guidelines. Evidence from China also points to the continuing need for school-based physical activity promotion. The China 2022 Report Card on Physical Activity for Children and Adolescents indicated that youth physical activity indicators remained far from ideal, with organized sport participation receiving a particularly low grade (Liu et al., 2023). Within schools, physical education (PE) is especially important because it provides a structured curricular context in which students can develop physical competence, experience movement with peers, and build dispositions toward lifelong physical activity. The Quality Physical Education framework emphasizes equality, safeguarding, inclusion, and meaningful participation as central principles of PE provision (McLennan and Thompson, 2015).

Within this public health context, exercise intention deserves closer scholarly attention. According to the Theory of Planned Behavior, intention is the most immediate motivational antecedent of planned behavior and reflects the degree to which individuals are willing to perform a given action (Ajzen, 1991). In the physical activity domain, meta-analytic evidence has supported the central role of intention in explaining exercise-related behavior (Hagger et al., 2002). Research involving Chinese junior school students has also shown that exercise intention is positively related to exercise behavior, suggesting that stronger intention provides an important psychological basis for actual participation in physical exercise (Chen et al., 2022). In the present study, Exercise Intention (EI) refers to general willingness and planning to engage in regular physical exercise rather than a PE-specific measure of lesson attendance, in-class engagement, or physical activity accumulated during PE. This distinction is important. For junior middle school students, however, school is a major setting in which exercise intentions can be expressed through compulsory PE lessons, school fitness testing, recess and active breaks, extracurricular sport, and other organized movement opportunities. General EI can therefore be understood as a form of motivational readiness that may support participation across school movement contexts, while not being equivalent to PE participation itself. Consistent with the social relevance of PE, a large cross-national study found that participation in PE classes was associated with lower social isolation among adolescents (Araujo et al., 2024).

Much existing research on adolescent exercise intention has focused on attitudes, perceived behavioral control, self-efficacy, family support, school resources, and health knowledge (Balla et al., 2024; de Oliveira Barbosa et al., 2024; Lima et al., 2023). These factors are important, yet they do not fully capture the subtle interpersonal pressures embedded in everyday school life. For Chinese adolescents, the school is a central setting for academic learning, peer relationship formation, identity development, and social evaluation (Shao and Kang, 2022). Peer relationships become especially salient during adolescence, when young people become more sensitive to acceptance, reputation, and belonging within peer groups (Brown and Larson, 2009). These dynamics are particularly relevant to PE and other school movement contexts because participation is public, bodily, and often organized through pairs, teams, group tasks, and performance comparisons. Recent review evidence indicates that peer relationships in PE can support positive developmental outcomes but can also amplify exclusion and isolation (Fassaert et al., 2026). Negative interpersonal experiences in school may therefore extend beyond emotional distress and become linked to students’ willingness to participate in visible and socially embedded physical activity. This study focuses on one underexamined form of negative school social experience: negative school gossip.

### 1.2 Negative school gossip and exercise intention

Gossip generally refers to evaluative communication about an absent third party and often involves the circulation of reputation-related information within social groups (Foster, 2004). Although gossip can serve social functions such as norm regulation and information exchange, negative gossip may become harmful when it conveys unfavorable, damaging, or false information about a target person. In the school context, Negative School Gossip (NSG) refers to students’ perceived experience of being the target of negative comments, rumors, or reputation-damaging information spread by teachers or classmates (Shen et al., 2024; Xiong et al., 2023). Prior research has treated NSG as a form of relational aggression because it operates through indirect social evaluation rather than direct confrontation (Crick and Grotpeter, 1995; Xiong et al., 2023).

Compared with overt conflict, NSG may be particularly harmful for adolescents because it is indirect, difficult to verify, and highly connected to reputation. When negative information circulates behind a student’s back, the target may not know who has heard it, whether others believe it, or when it will reappear in future interactions. This ambiguity may intensify social-evaluative threat. Research on Chinese adolescents has linked NSG with maladaptive psychological and behavioral outcomes. Wang et al. (2020) found that NSG was associated with suicide intention among Chinese junior high school students through academic burnout. Xiong et al. (2023) reported that NSG was associated with mobile phone addiction among Chinese adolescents through anxiety and experiential avoidance. Shen et al. (2024) further showed that NSG was negatively associated with prosocial behavior among Chinese high school students through basic psychological needs satisfaction. These studies suggest that NSG is more than casual talk; it may function as a school-based interpersonal stressor with implications for adolescent adjustment.

The potential relevance of NSG to exercise intention is especially clear in school movement settings. PE lessons and related school physical activity often involve bodily exposure, skill comparison, performance evaluation, group selection, and peer observation (Åsebø et al., 2022; Haug et al., 2023). From an inclusion perspective, gossip may also serve as a marker of a peer climate in which a student perceives stigma, exclusion, or reduced relational standing. A student can have formal access to the same PE lesson as classmates yet still feel that visible participation carries a reputational cost if mistakes, body appearance, or performance may become material for ridicule or further gossip. Thus, inclusive and equitable PE concerns not only access to movement opportunities but also whether students experience sufficient psychological safety to attempt, fail, learn, and be seen without humiliation.

In this sense, NSG is not simply a background interpersonal problem. It may correspond with a social-evaluative climate in which public exercise feels more threatening or less attractive. Under Self-Determination Theory, climates associated with lower relatedness, competence, or autonomy are expected to covary with weaker self-determined motivation (Ryan and Deci, 2000). In the present study, this rationale motivates an associational hypothesis rather than a causal claim:

*H1*: Negative School Gossip is negatively associated with Exercise Intention among Chinese adolescents.

### 1.3 Self-esteem as a psychological bridge between negative school gossip and exercise intention

Self-Esteem (SE) refers to an individual’s overall evaluation of self-worth, self-acceptance, and personal value (Rosenberg, 1965). During adolescence, SE is closely tied to social feedback because adolescents are actively constructing their self-concept while becoming more attentive to peer approval and group belonging (Pan et al., 2024; Mullan et al., 2023). Sociometer theory offers a strong account of this correspondence. According to Leary et al. (1995), self-esteem functions as a broad internal indicator of perceived social acceptance and relational value. In this framework, higher perceived acceptance tends to co-occur with higher SE, whereas rejection, devaluation, or exclusion tends to co-occur with lower SE (Van Schie et al., 2023; Mullan et al., 2023). Cross-sectional data cannot show that a specific episode of gossip first activates a sociometer and subsequently changes SE; rather, they can test whether perceived NSG and SE covary in the direction predicted by sociometer theory.

NSG is theoretically relevant to the sociometer because it concerns reputation-related social judgment. Perceiving unfavorable information circulating at school may coincide with a sense of lower acceptance, belonging, or relational value. For adolescents, such signals may be particularly salient in stable peer groups in which reputation is repeatedly visible. Broader peer-victimization evidence supports an association between adverse peer experiences and lower SE (Mullan et al., 2023), while NSG research has linked gossip with maladaptive adjustment and lower basic psychological need satisfaction (Shen et al., 2024; Xiong et al., 2023). On this basis, NSG is expected to be negatively associated with SE.

SE may also be positively associated with EI. Adolescents with higher global SE may feel more comfortable entering achievement-oriented, peer-observed, and physically expressive settings, including PE and school sport, where performance and self-presentation are salient. Physical activity is associated with self-related perceptions, and school- or gymnasium-based activity interventions have been linked to self-esteem and self-concept outcomes (Liu et al., 2015). The present study, however, measures intention rather than behavior; the relevant expectation is therefore a positive cross-sectional association between SE and willingness to exercise.

Taken together, sociometer theory motivates a cross-sectional indirect-association model: greater perceived NSG is expected to co-occur with lower SE, and lower SE is expected to co-occur with weaker EI. If the product of these two modeled associations is statistically different from zero, it would be consistent with a sociometer-based account in which global self-worth captures part of the covariance between reputation-related peer experiences and exercise motivation. It would not, however, demonstrate that NSG temporally changes SE or that SE causally transmits the association to EI. The present study therefore proposes the following hypotheses:

*H2*: Negative School Gossip is negatively associated with Self-Esteem among Chinese adolescents.

*H3*: Negative School Gossip has a statistically significant indirect association with Exercise Intention through Self-Esteem among Chinese adolescents.

### 1.4 The present study and theoretical model

The present study examines the associations among Negative School Gossip, Self-Esteem, and Exercise Intention in a sample of Chinese adolescents. It addresses a gap in adolescent physical activity research by moving beyond individual-level health beliefs to consider reputation-related school social experiences. Because EI is general rather than PE-specific, the study does not test participation in PE lessons directly. Instead, it uses PE and school movement settings as the principal contextual domain in which the theoretical relevance of social visibility, peer evaluation, and inclusion can be articulated.

The central theoretical contribution is not simply adding a new antecedent or outcome. Rather, the study asks whether a sociometer-consistent pattern is observable in an achievement-oriented, peer-observed, and physically demanding domain. If NSG is associated with lower SE and SE with lower EI, this would suggest that adolescents’ global monitor of relational value is relevant to motivation for entering visible physical activity settings. At the same time, a remaining direct NSG-EI association would qualify a purely sociometer-based account by indicating that global SE captures only part of the covariance; exercise-specific constructs such as social physique anxiety, physical self-concept, perceived competence, or fear of negative evaluation may also be relevant. A second contribution is to connect reputation-based exclusion and stigma with inclusive PE: the model treats peer climate and psychological safety as plausible contextual conditions for equitable willingness to participate. These are conceptual implications of cross-sectional associations, not demonstrated causal mechanisms.

Based on the above reasoning, the structural model specifies simultaneous cross-sectional associations from NSG to SE and EI and from SE to EI, together with the resulting indirect association through SE. Figure 1 presents this hypothesized statistical model.

**Figure 1:**
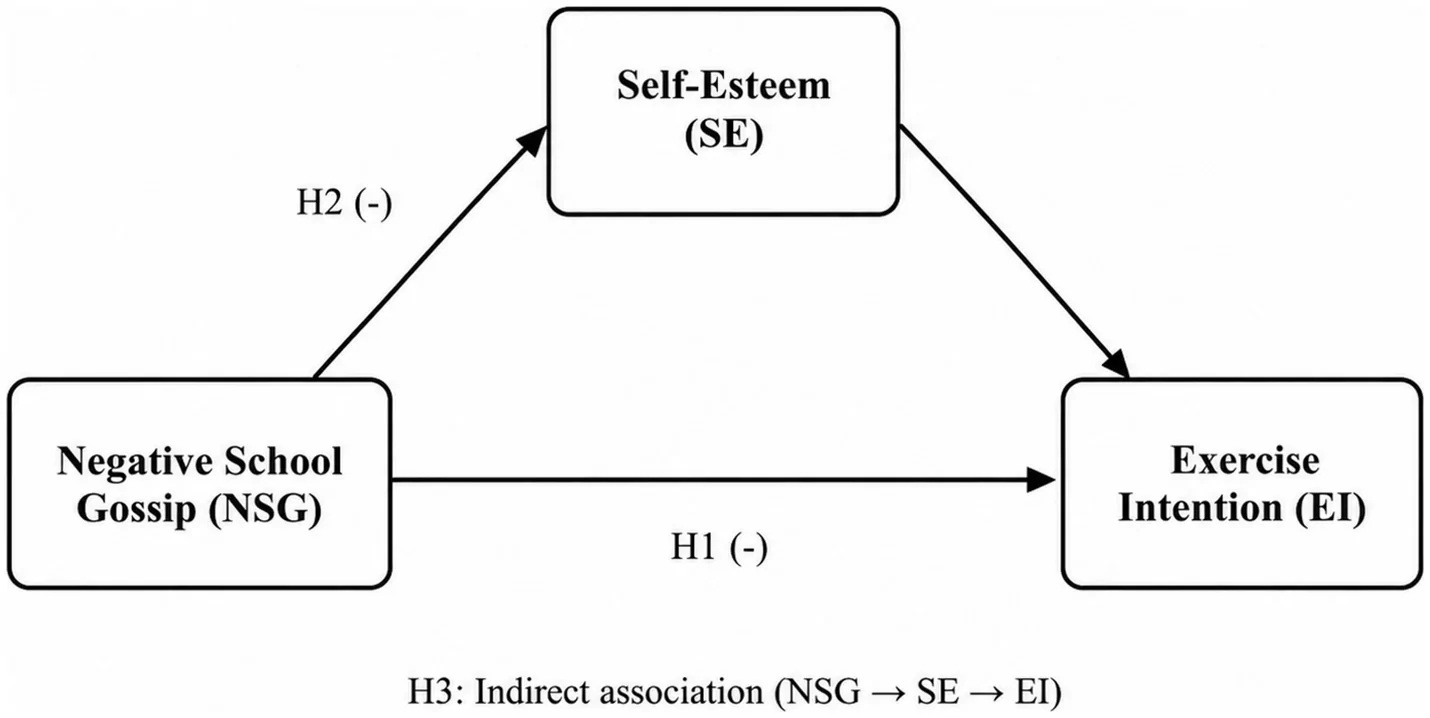
Hypothesized cross-sectional association model among negative school gossip, self-esteem, and exercise intention.

## 2 Methods

### 2.1 Participants and procedure

This study adopted a cross-sectional questionnaire design to examine the associations among negative school gossip, self-esteem, and exercise intention among junior middle school students in Chengdu, Sichuan Province, and Chongqing Municipality, China. Participants were recruited through a multistage sampling procedure. First, five junior middle schools were randomly selected from Chengdu and five from Chongqing, yielding 10 participating schools. Second, one class was selected from each of Grades 7, 8, and 9 in each school, yielding 30 classes in total. To reduce potential teacher overlap and cross-class influence, only one class per grade was included from each school. Sixty students were recruited from each selected class, resulting in an initial sample of 1,800 students (10 schools × 3 classes per school × 60 students per class). The target population comprised students in Grades 7–9, a developmental period characterized by frequent peer interaction, heightened sensitivity to social evaluation, and rapid formation of self-concept. Students were eligible if they were enrolled in Grades 7–9, were able to understand and complete the questionnaire independently, and voluntarily agreed to participate. Responses were excluded if they met any of the predefined invalid-response criteria described below. The initial allocation was equal across schools, with 180 students recruited from each school (60 students from each of three grade-specific classes). By design, the data had a hierarchical structure, with students nested within 30 classes and classes nested within 10 schools.

Data were collected between January 5, 2026 and April 30, 2026 through Wenjuanxing, a widely used online survey platform in China. Before data collection, the research team contacted school administrators and classroom teachers to explain the research purpose, questionnaire procedure, confidentiality arrangements, and participation requirements. After permission was obtained from the participating schools, the questionnaire link was distributed to students through class-based online channels under the coordination of teachers. Participants completed the questionnaire anonymously using their own electronic devices or school-arranged devices. The online questionnaire was configured so that respondents could not submit the survey if any required item was left unanswered; therefore, no item-level missing data were present in the submitted questionnaires.

To improve data quality, one attention-check item was embedded in the questionnaire: “To show that you are reading the items carefully, please select ‘Agree’ for this item”. Responses were screened according to predefined criteria: (1) failure to pass the attention-check item; (2) a completion time of less than 1 min; and (3) highly regular or completely identical response patterns, defined as response strings that were identical across substantive scale items or showed an obvious repetitive pattern. A total of 1,800 questionnaires were submitted. Eighty-seven participants met at least one invalid-response criterion and were excluded, leaving 1,713 valid questionnaires for analysis, corresponding to a valid-response rate of 95.17%. Because the online survey required a response to every item before submission, no item-level missing data were present in the submitted questionnaires.

All procedures involving human participants were reviewed and approved by the Ethics Committee of the School of Sports Training, Chengdu Sport University. Participation was voluntary and anonymous. Before completing the survey, participants were informed of the study purpose, the approximate time required, the confidentiality of their responses, and their right to withdraw at any time without penalty. Informed consent was obtained from the participants and their legal guardians before data collection.

Table 1 presents the demographic characteristics of the final sample. Among the 1,713 participants, 880 were male (51.37%) and 833 were female (48.63%). The sample included 594 Grade 7 students (34.68%), 589 Grade 8 students (34.38%), and 530 Grade 9 students (30.94%). In terms of region, 762 students were from urban areas (44.48%), 566 from suburban areas (33.04%), and 385 from rural areas (22.48%). Regarding parental education, 514 students reported middle school education or below for parental education (30.01%), 633 reported high school education (36.95%), 466 reported college education (27.20%), and 100 reported graduate education (5.84%). In addition, 658 students were only children (38.41%), while 1,055 were not only children (61.59%). For family economic status, 310 students reported low family economic status (18.10%), 1,130 reported middle family economic status (65.97%), and 273 reported high family economic status (15.94%).

**Table 1:** Demographic characteristics of participants (*N* = 1,713).

Characteristic	Category	*n*	%
Gender	Male	880	51.37
	Female	833	48.63
Grade	Grade 7	594	34.68
	Grade 8	589	34.38
	Grade 9	530	30.94
Region	Urban	762	44.48
	Suburban	566	33.04
	Rural	385	22.48
Parental education	Middle school or below	514	30.01
	High school	633	36.95
	College	466	27.20
	Graduate	100	5.84
Only child	Yes	658	38.41
	No	1,055	61.59
Family economic status	Low	310	18.10
	Middle	1,130	65.97
	High	273	15.94

### 2.2 Measures

All constructs were assessed using established self-report scales. To maintain consistency in response format and reduce unnecessary cognitive burden for adolescent respondents, all measures were administered using a five-point Likert-type format. For Negative School Gossip, response options ranged from 1 = never to 5 = always. For Exercise Intention and Self-Esteem, response options ranged from 1 = strongly disagree to 5 = strongly agree. Mean scores were calculated for each construct, with higher scores indicating higher levels of the corresponding variable. Negatively worded items in the self-esteem measure were reverse-coded before computing the scale score. All three constructs were modeled as first-order reflective latent variables in the subsequent structural equation modeling analyses. Internal consistency was assessed using Cronbach’s alpha and composite reliability, whereas convergent validity and discriminant validity were evaluated through standardized factor loadings, average variance extracted, and inter-construct correlations.

#### 2.2.1 Negative school gossip

Negative School Gossip (NSG) refers to students’ perceived experience of being the target of unfavorable, damaging, or false information spread by teachers or classmates in the school context. NSG was measured using a three-item adaptation of the Negative School Gossip Scale attributed to Wang et al. (2020), which has been applied in studies of Chinese adolescents (Shen et al., 2024; Xiong et al., 2023). The scale assessed the frequency with which student’s perceived negative gossip directed at them at school. A representative item is: “Someone at school spread rumors about me”. Participants rated each item on a five-point frequency scale ranging from 1 = never to 5 = always. The mean score of the three items was used, with higher scores indicating more frequent perceived negative school gossip. This brief operationalization captures generalized perceived gossip victimization at school; it does not distinguish gossip source (e.g., classmate versus teacher), content domain (e.g., academic performance, appearance, personality, peer relationships, or exercise performance), perceived audience, or circulation scope. Accordingly, NSG in the present study should be interpreted as a generic school-context interpersonal exposure rather than an exercise-specific gossip measure.

#### 2.2.2 Exercise intention

Exercise Intention (EI) refers to students’ willingness and intention to engage in physical exercise in the near future. EI was assessed using a three-item Exercise Intention Scale developed within the framework of the Theory of Planned Behavior (Ajzen, 1991, 2006) and previously used among Chinese junior school students (Chen et al., 2022). All three items referred to engaging in physical exercise for more than 20 min at least three times per week during the following 2 weeks. The items were: (1) “In the next two weeks, I plan to take physical exercise for more than 20 minutes at least three times a week”; (2) “In the next two weeks, I intend to take physical exercise for more than 20 minutes at least three times a week”; and (3) “In the next two weeks, I hope to take physical exercise for more than 20 minutes at least three times a week”. Participants rated each item on a five-point Likert-type scale ranging from 1 = strongly disagree to 5 = strongly agree. The original version reported by Chen et al. (2022) used a seven-point response format; therefore, the five-point format used in the present study represents an adaptation of the response scale. The mean score of the three items was calculated, with higher scores indicating stronger exercise intention. Importantly, the items assess general exercise intention rather than intention to participate specifically in PE lessons, school sport, recess activity, or other school-based movement settings. Accordingly, EI should be interpreted as general exercise intention, and its implications for PE participation are contextual rather than direct measurement claims.

#### 2.2.3 Self-esteem

Self-Esteem (SE) refers to an individual’s overall evaluation of self-worth, self-acceptance, and personal value. SE was assessed using the Rosenberg Self-Esteem Scale (RSES), a widely used instrument for measuring global self-esteem (Rosenberg, 1965). The Chinese version of the RSES has been examined in Chinese samples and has shown acceptable psychometric evidence for assessing self-esteem in the Chinese context (Jiang et al., 2023). The scale contained 10 items, including positively and negatively worded statements. A representative item is: “On the whole, I am satisfied with myself.” Participants rated each item on a five-point Likert-type scale ranging from 1 = strongly disagree to 5 = strongly agree. Negatively worded items were reverse-coded before analysis. The mean score of the 10 items was then calculated, with higher scores indicating higher global self-esteem. In the present study, SE was treated as a positive psychological resource and mediator.

### 2.3 Data analysis

All statistical analyses were conducted using Mplus 8.3 (Muthén and Muthén, 2019). Because all focal scale items used five response categories and the sample size was large, the item-level indicators were treated as approximately continuous and estimated using maximum likelihood estimation (Rhemtulla et al., 2012). All focal constructs were modeled as first-order reflective latent variables with their corresponding item-level indicators. Standardized estimates were reported using the STDYX metric. Statistical significance was evaluated at *p* < 0.05.

The analysis proceeded in four steps, corresponding to the order of the results section. First, preliminary analyses were conducted to examine data quality and potential common method bias. Because the online questionnaire was configured to prevent submission when required items were left unanswered, no item-level missing data were present in the final dataset. To reduce common method bias, several procedural remedies were adopted during data collection, including anonymous responses, clear instructions, separation of measures into different sections, different response anchors across measures, and an attention-check item. These procedures were consistent with recommendations for reducing common method variance in self-report survey research (Podsakoff et al., 2003). Common method bias was further examined using confirmatory factor analysis by comparing alternative measurement models. Specifically, a one-factor model, three two-factor models, the hypothesized three-factor model, and an unmeasured latent method construct model were estimated. If the one-factor model showed poor fit and the inclusion of an unmeasured method factor did not substantially improve model fit, common method bias was considered unlikely to dominate the substantive associations. These checks address measurement-source covariance only; they cannot establish temporal order, exclude reverse causality, or convert cross-sectional associations into causal estimates.

Second, descriptive statistics, reliability, and validity were examined. Means and standard deviations were calculated for the scale scores of negative school gossip, self-esteem, and exercise intention. Internal consistency was evaluated using Cronbach’s alpha, with values of 0.70 or higher considered acceptable for research purposes (Nunnally and Bernstein, 1994). Composite reliability (CR) and average variance extracted (AVE) were calculated to assess construct reliability and convergent validity. CR values of 0.70 or above and AVE values of 0.50 or above were regarded as acceptable evidence of reliability and convergent validity (Fornell and Larcker, 1981). Standardized factor loadings were also inspected, with higher loadings indicating stronger representation of the latent construct by its observed indicators. Discriminant validity was examined using the Fornell–Larcker criterion and the heterotrait–monotrait ratio (HTMT). Under the Fornell–Larcker criterion, the square root of AVE for each construct should exceed its correlations with other constructs (Fornell and Larcker, 1981). HTMT values below 0.85 were taken as evidence of adequate discriminant validity under a conservative criterion (Henseler et al., 2015).

Third, the hypothesized latent-variable structural equation model was estimated to examine the proposed cross-sectional indirect-association framework. Model fit was evaluated using multiple indices, including the chi-square statistic, degrees of freedom, *χ*^2^/df, comparative fit index (CFI), Tucker-Lewis index (TLI), root mean square error of approximation (RMSEA), and standardized root mean square residual (SRMR). Following commonly used SEM guidelines, CFI and TLI values of 0.90 or above were considered acceptable and values of 0.95 or above indicated good fit; RMSEA and SRMR values of 0.08 or below were considered acceptable, with lower values indicating better fit (Hu and Bentler, 1999; Kline, 2023). The modeled regression associations from negative school gossip to self-esteem, from self-esteem to exercise intention, and from negative school gossip to exercise intention were estimated simultaneously. Standardized coefficients, standard errors, z values, and *p* values were reported.

The indirect association was examined using bias-corrected bootstrap confidence intervals with 2,000 bootstrap samples. Bootstrap procedures are useful for estimating indirect associations because the sampling distribution of product terms is often asymmetric (Preacher and Hayes, 2008). The indirect association was considered statistically significant when the 95% bias-corrected bootstrap confidence interval did not include zero. Direct, indirect, and total associations were reported using STDYX-standardized estimates. Consistent with recommendations to avoid overinterpreting ratio-based mediation indices, the indirect-to-total ratio was retained only as a descriptive decomposition and was not treated as a primary or inferential effect-size measure (Preacher and Kelley, 2011). Interpretation therefore emphasized the standardized indirect association and its bootstrap confidence interval. Because all three focal constructs were measured at the same wave, the indirect estimate was treated as a statistical decomposition of modeled covariance. No temporal sequence or causal mediation was inferred, consistent with cautions about cross-sectional mediation (Maxwell and Cole, 2007; Maxwell et al., 2011).

Fourth, a sensitivity analysis was conducted to examine whether the main pattern remained stable after accounting for demographic covariates. Gender, grade, region, parental education, only-child status, and family economic status were entered as observed covariates in the latent-variable structural equation model. Parental education comprised four ordered categories (middle school or below, high school, college, and graduate education) and was coded 1–4 in ascending order of educational attainment. For this sensitivity analysis, the resulting score was entered as a single non-focal ordered covariate. Assigning ordered scores to an ordinal explanatory variable is a recognized parsimonious approach when the category ordering is substantively meaningful (Agresti, 2010). We acknowledge, however, that this coding imposes a linear trend with respect to the assigned scores and does not estimate separate category-specific effects. Accordingly, the parental-education coefficient was not interpreted substantively; the sensitivity analysis was used only to assess whether demographic adjustment altered the focal NSG-SE-EI association pattern. The covariate-adjusted estimates were compared with those from the main model, focusing on the consistency of the direction, magnitude, statistical significance, and bootstrap confidence intervals of the key direct, indirect, and total associations.

## 3 Results

### 3.1 Preliminary analyses, common method variance, and measurement model evaluation

Several procedural strategies were implemented to reduce potential common method variance. The questionnaire was administered anonymously, participants were informed that there were no right or wrong answers, and they were encouraged to respond according to their actual experiences. The focal measures were presented in separate sections, and different response anchors were used for the frequency-based negative school gossip items and the agreement-based self-esteem and exercise intention items. An attention-check item was also included to identify potentially careless responding.

Common method variance was further evaluated by comparing a series of alternative measurement models. As shown in Table 2, the one-factor model demonstrated poor fit, indicating that the observed indicators could not be adequately represented by a single general factor. The three alternative two-factor models also showed substantially poorer fit than the hypothesized three-factor model. In contrast, the three-factor model, in which negative school gossip, self-esteem, and exercise intention were specified as distinct latent constructs, showed excellent fit to the data, *χ*^2^(101) = 104.36, *χ*^2^/df = 1.03, CFI = 1.000, TLI = 1.000, RMSEA = 0.004, and SRMR = 0.013. Adding an unmeasured latent method construct produced virtually no improvement in model fit. Taken together, these results did not suggest that a single common factor or the modeled method factor accounted for a substantial proportion of the covariance among the focal measures. Nevertheless, these analyses cannot rule out all forms of common method variance inherent in a same-source, cross-sectional self-report design.

**Table 2:** Comparison of alternative measurement models for common method bias.

Model	Model specification	*χ* ^2^	df	*χ*^2^/df	CFI	TLI	RMSEA	SRMR
Model 1	One-factor model: all indicators loaded on a single latent factor	3683.12	104	35.41	0.771	0.735	0.142	0.116
Model 2A	Two-factor model A: NSG + SE; EI	2299.95	103	22.33	0.859	0.836	0.112	0.090
Model 2B	Two-factor model B: NSG + EI; SE	1643.92	103	15.96	0.901	0.885	0.093	0.098
Model 2C	Two-factor model C: SE + EI; NSG	1492.72	103	14.49	0.911	0.896	0.089	0.074
Model 3	Three-factor model: NSG; SE; EI	104.36	101	1.03	1.000	1.000	0.004	0.013
Model 4	ULMC model: three substantive factors plus one latent common method factor	104.36	100	1.04	1.000	1.000	0.005	0.013

NSG, Negative school gossip; SE, Self-esteem; EI, Exercise intention; ULMC, Unmeasured latent method construct. Models were estimated using item-level indicators.

The identical global fit statistics subsequently obtained for the structural model reflect the parameterization of the models rather than duplication of the measurement-model results. The three-factor measurement model and the structural model retained the same item-level measurement specification. In the measurement model, the latent factors representing negative school gossip, self-esteem, and exercise intention were freely correlated. In the structural model, these latent relations were reparameterized as the recursive paths NSG → SE, NSG → EI, and SE → EI, with the residual variances of the endogenous latent variables freely estimated. For three latent variables under this specification, the structural portion of the model is just identified and can reproduce the same latent covariance structure as the freely correlated three-factor model. Therefore, the structural reparameterization introduces no additional overidentifying restrictions, and the model degrees of freedom and global fit indices remain unchanged. Accordingly, the identical *χ*^2^, CFI, TLI, RMSEA, and SRMR values are expected under this specification.

### 3.2 Descriptive statistics, reliability, and validity

Table 3 presents the descriptive statistics and measurement properties of the study variables. The mean scores of the three focal constructs were close to the midpoint of the five-point scale, indicating sufficient variability in participants’ responses. The internal consistency estimates were acceptable to excellent, with Cronbach’s alpha values ranging from 0.788 to 0.941. Composite reliability values were also above the commonly used threshold of 0.70, and the average variance extracted values exceeded 0.50 for all constructs. The standardized factor loadings were all within acceptable ranges, supporting the convergent validity of the measurement model.

**Table 3:** Descriptive statistics and measurement properties of study variables.

Variable	Mean	SD	*α*	Std. loading range	CR	AVE
NSG	2.990	1.233	0.849	0.757–0.863	0.867	0.686
SE	3.011	1.147	0.941	0.731–0.863	0.949	0.650
EI	3.005	1.195	0.788	0.738–0.796	0.817	0.598

NSG, Negative school gossip; SE, Self-esteem; EI, Exercise intention. Means and standard deviations are based on scale scores. *α*, Cronbach’s alpha; CR, Composite reliability; AVE, Average variance extracted.

The bivariate associations among the focal variables are shown in Table 4. Negative school gossip was negatively correlated with both self-esteem and exercise intention, whereas self-esteem was positively correlated with exercise intention. These associations were in the expected directions and provided preliminary support for the proposed mediation model. The square roots of AVE, reported on the diagonal, were higher than the corresponding inter-construct correlations, suggesting acceptable discriminant validity.

**Table 4:** Correlations and discriminant validity among study variables.

Variable	NSG	SE	EI
NSG	0.828		
SE	−0.140***	0.806	
EI	−0.081***	0.214***	0.773

NSG, Negative school gossip; SE, Self-esteem; EI, Exercise intention. Values on the diagonal represent the square roots of AVE. Off-diagonal values represent Pearson correlations based on scale scores. ***p < 0.001.

Discriminant validity was also evaluated using the heterotrait–monotrait ratio. As presented in Table 5, all HTMT values were well below the conservative threshold of 0.85, further supporting the empirical distinctiveness of the three constructs.

**Table 5:** Discriminant validity based on the heterotrait–monotrait ratio.

Construct	NSG	SE	EI
NSG	–	–	–
SE	0.157	–	–
EI	0.099	0.249	–

NSG, Negative school gossip; SE, Self-esteem; EI, Exercise intention. Values below the diagonal represent HTMT values computed from item-level indicators.

### 3.3 Structural model and hypothesis testing

The hypothesized latent-variable structural equation model was estimated using the same item-level measurement specification as the three-factor measurement model. As explained in Section 3.1, the structural model yielded identical global fit indices because the structural portion was just identified under the present recursive specification: *χ*^2^(101) = 104.36, *χ*^2^/df = 1.03, CFI = 1.000, TLI = 1.000, RMSEA = 0.004, and SRMR = 0.013. Accordingly, hypothesis testing focused on the estimated structural coefficients. As shown in Figure 2 and Table 6, NSG was negatively associated with EI (*β* = −0.063, SE = 0.029, *z* = −2.160, *p* = 0.031), consistent with H1, although the magnitude of this association was small. NSG was also negatively associated with SE (*β* = −0.157, SE = 0.027, *z* = −5.820, *p* < 0.001), while SE was positively associated with EI (*β* = 0.240, SE = 0.027, *z* = 8.978, *p* < 0.001), supporting H2.

**Figure 2:**
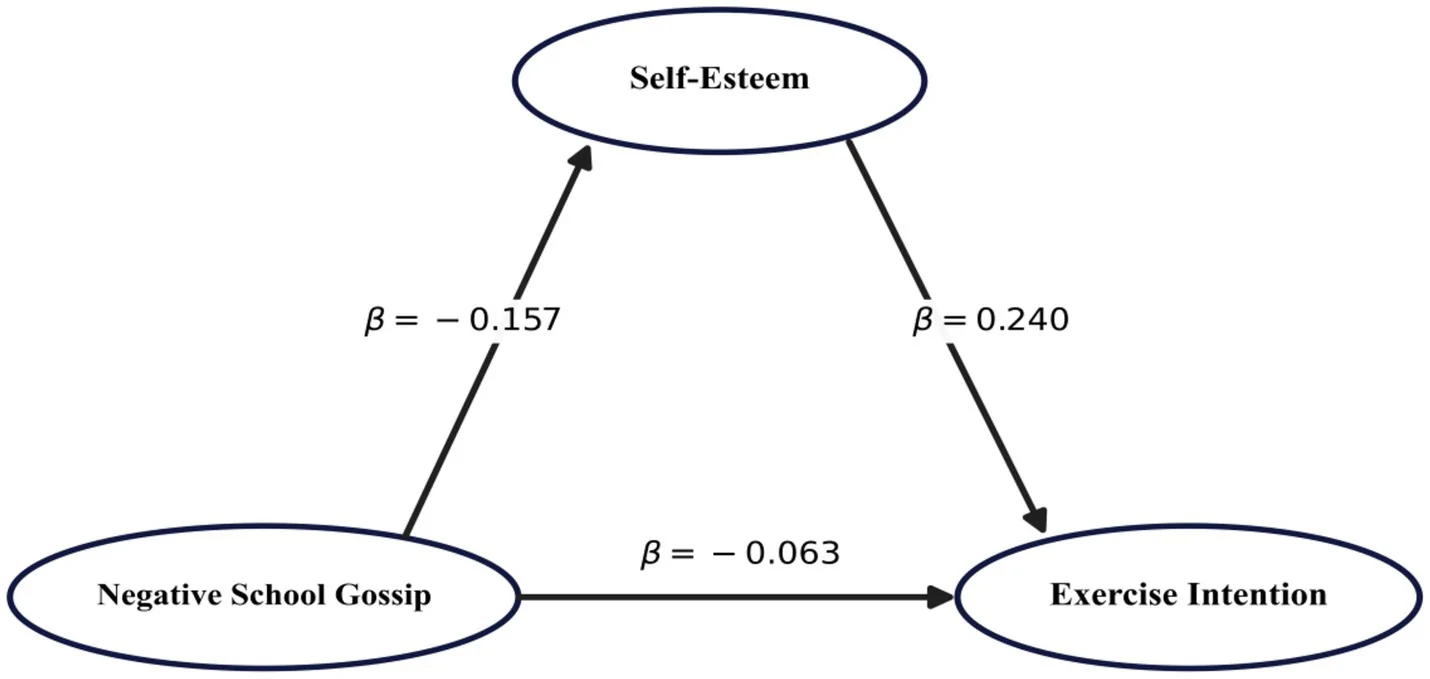
Standardized coefficients from the cross-sectional structural model.

**Table 6:** Standardized structural path coefficients of the main latent-variable SEM.

Path	Standardized coefficient (β)	SE	*z*	*p*
NSG → SE	−0.157	0.027	−5.820	< 0.001
SE → EI	0.240	0.027	8.978	< 0.001
NSG → EI	−0.063	0.029	−2.160	0.031

NSG, Negative school gossip; SE, Self-esteem; EI, Exercise intention. The main model was estimated with item-level indicators and did not include demographic covariates. z = estimate/SE as reported by Mplus.

Bias-corrected bootstrap analysis with 2,000 samples further showed a significant indirect association between NSG and EI through SE [*β* = −0.038, Boot SE = 0.008, 95% CI (−0.055, −0.024)], supporting H3 (Table 7). The total standardized association was *β* = −0.101 [95% CI (−0.162, −0.047)], with the indirect component accounting for 37.62% of the total association. Because the data were cross-sectional, these estimates represent concurrent statistical associations rather than causal or temporal effects.

**Table 7:** Bias-corrected bootstrap results for standardized direct and indirect associations in the main latent-variable SEM.

Path	*β*	Boot SE	Boot LLCI	Boot ULCI	Proportion of total standardized association (%)
Direct association: NSG → EI	−0.063	0.029	−0.121	−0.005	62.38
Indirect association: NSG → SE → EI	−0.038	0.008	−0.055	−0.024	37.62
Total association: NSG → EI	−0.101	0.029	−0.162	−0.047	100.00

NSG, Negative school gossip; SE, Self-esteem; EI, Exercise intention. Effects are STDYX-standardized estimates from Mplus MODEL INDIRECT with 2,000 bootstrap samples. Boot SE, bootstrap standard error; Boot LLCI/ULCI = lower/upper limit of the 95% bias-corrected bootstrap confidence interval.

Bias-corrected bootstrap analysis with 2,000 bootstrap samples was conducted to examine the standardized indirect association. As shown in Table 7, Hypothesis 3 specified a statistically significant indirect association between NSG and EI through SE, and this hypothesis was supported. The standardized indirect association was *β* = −0.038 (Boot SE = 0.008), with a 95% bias-corrected bootstrap confidence interval of [−0.055, −0.024]. The total standardized association between NSG and EI was also negative [*β* = −0.101, 95% bootstrap CI (−0.162, −0.047)]. The fitted model therefore contained both a direct association component and an indirect component through SE. The direct association represented 62.38% of the total standardized association, whereas the indirect association represented 37.62%. Thus, the SE-related component was meaningful but not the larger component. These proportions describe the decomposition of a cross-sectional standardized association; they do not indicate the percentage of variance in EI explained, nor do they establish a temporal or causal mechanism.

### 3.4 Sensitivity analysis with demographic covariates

A sensitivity analysis was conducted by adding demographic covariates to the latent-variable SEM. The covariates included gender, grade, region, parental education, only-child status, and family economic status. As described in Section 2.3, the four-category parental education variable was coded from 1 to 4 in ascending order and entered as an ordered-score covariate solely for demographic adjustment; its coefficient was not a target of substantive inference. The purpose of this analysis was to examine whether the main NSG-SE-EI association pattern remained stable after accounting for basic demographic characteristics.

As shown in Table 8, the standardized estimates in the covariate-adjusted model were highly similar to those in the main model. The negative NSG-SE association, the positive SE-EI association, and the direct NSG-EI association retained the same directions and remained statistically significant. The standardized indirect association through SE was unchanged in magnitude, and its bootstrap confidence interval again excluded zero. These findings indicate that the cross-sectional direct and indirect association pattern was stable to adjustment for the selected demographic covariates.

**Table 8:** Sensitivity analysis controlling for demographic covariates in the latent-variable SEM.

Effect/path	Main estimate	Sensitivity estimate	Sensitivity p	Sensitivity boot LLCI	Sensitivity boot ULCI	Metric
NSG → SE	−0.157	−0.157	< 0.001	–	–	STDYX β
SE → EI	0.240	0.244	< 0.001	–	–	STDYX β
NSG → EI	−0.063	−0.066	0.024	–	–	STDYX β
NSG → SE → EI	−0.038	−0.038	< 0.001	−0.055	−0.024	STDYX β
Total NSG → EI	−0.101	−0.104	< 0.001	−0.165	−0.050	STDYX β

NSG, Negative school gossip; SE, Self-esteem; EI, Exercise intention. The sensitivity model controlled for gender, grade, region, parental education, only-child status, and family economic status. Parental education comprised four ordered categories and was coded 1–4 in ascending order; this single-score coding estimates an approximately linear ordinal trend and was used only for covariate adjustment. The main model did not include demographic covariates.

## 4 Discussion

### 4.1 Negative school gossip, inclusion, and PE-relevant social-evaluative contexts

This study examined concurrent associations among Negative School Gossip (NSG), Self-Esteem (SE), and Exercise Intention (EI) among Chinese adolescents. The outcome was general EI, not PE-specific intention or behavior, so the findings should not be read as direct evidence that gossip changes PE participation. Their relevance to PE lies in the school context in which exercise motivation is expressed: PE lessons, fitness tests, class exercises, playground activity, and school sport are visible, interactive, and often evaluative. In such settings, willingness to move may be associated not only with health beliefs and competence, but also with whether students expect respectful treatment and feel sufficiently safe to participate in front of peers.

From an inclusion perspective, NSG is relevant because reputation-damaging communication can coexist with exclusion, stigma, and a peer climate in which relational value feels uncertain. Inclusive PE requires more than identical formal access to activities; equitable participation is also difficult when students anticipate ridicule, public comparison, or reputational exposure. The observed negative NSG-EI association is therefore consistent with the possibility that students who perceive a less accepting peer climate also report lower willingness to exercise. This is an inclusion-related interpretation of the association, not evidence that gossip caused non-participation.

Adolescence may make this correspondence especially salient because peer evaluation, reputation, acceptance, and exclusion are developmentally prominent (Somerville, 2013). NSG is indirect and ambiguous: targets may not know who has heard information or how it will reappear in later interactions. PE and other school movement contexts can amplify visibility through bodily display, skill comparison, group selection, and performance in front of classmates (Åsebø et al., 2022; Haug et al., 2023). The present association therefore identifies reputation safety and peer climate as plausible social correlates of exercise motivation that merit PE-specific testing.

### 4.2 Self-esteem as a sociometer in peer-observed physical activity

The SE-related indirect association is theoretically informative when interpreted with appropriate cross-sectional restraint. Sociometer theory conceptualizes SE as an internal indicator of perceived acceptance and relational value (Leary et al., 1995). The observed pattern - higher NSG associated with lower SE, and higher SE associated with stronger EI - is consistent with this framework. In a peer-observed physical context, the sociometer may be especially relevant because students are not only completing a task; they are moving, being seen, comparing performance, and exposing bodily competence. The data cannot show that a sociometer “alarm” was triggered in time, but they show a covariance pattern compatible with that proposition.

This extends the sociometer question into an achievement-oriented and physically demanding domain. A general measure of self-worth may matter when adolescents decide whether they are willing to enter settings where success, failure, body movement, and peer evaluation are visible. Broader evidence linking peer victimization with lower SE (Mullan et al., 2023) and evidence on PE visibility (Åsebø et al., 2022) make this interpretation plausible. At the same time, the study does not establish that global SE is the only or most proximal self-evaluative construct operating in PE-relevant contexts.

Indeed, exercise-related settings may recruit domain-specific evaluations that are more proximal to visible movement than global SE, including physical self-concept, perceived physical competence, social physique anxiety, and fear of negative evaluation. Research has documented close links between youth physical activity and physical self-concept (Babic et al., 2014), while social physique anxiety directly concerns apprehension about others evaluating one’s physique (Hart et al., 1989). These constructs provide theoretically meaningful candidates for parallel or sequential indirect associations in future longitudinal models.

### 4.3 Direct and indirect components: a qualified sociometer account

The relative magnitudes of the modeled components help refine the theoretical and practical interpretation. In absolute standardized terms, the direct NSG-EI association was small (*β* = 0.063), and the indirect association through SE was also modest (*β* = 0.038). With a sample of 1,713 students, the direct coefficient reached statistical significance at *p* = 0.031, but statistical significance should not be conflated with a strong or practically large association. The total standardized association was *β* = −0.101. Thus, the results indicate a modest pattern of concurrent association rather than a large effect on exercise intention. The direct component represented 62.38% of the total standardized association and the SE-related indirect component represented 37.62%; these percentages describe decomposition of the modeled standardized association, not the amount of variance explained or the expected impact of an intervention. Practical implications should therefore be framed as hypotheses about potentially relevant peer-climate processes rather than as evidence that reducing gossip would produce a large change in exercise intention.

This pattern places a useful boundary around a simple sociometer explanation. Global SE captures a statistically detectable but modest portion of the covariance expected by sociometer theory, while considerable association remains outside that statistical component. One possibility is that reputation-related peer experiences are represented by multiple evaluative systems: a broad relational-value signal indexed by global SE and more context-specific concerns about bodily appearance, competence, belonging, or anticipated peer judgment. Another possibility is reciprocal or reverse ordering. Distinguishing these accounts will require repeated measures or experiments that compare global SE with domain-specific constructs such as social physique anxiety and physical self-concept.

### 4.4 Theoretical implications

These findings support three theoretical refinements. First, they broaden the domain in which sociometer theory can be examined. Prior applications emphasize social acceptance and self-worth; the present pattern suggests that relational-value concerns are also relevant to motivation for entering peer-observed physical activity settings. This is not a demonstration of a causal sociometer process, but it identifies PE-relevant exercise motivation as a theoretically productive context in which the sociometer’s operation can be tested more directly.

Second, the findings suggest a contextual boundary condition. School movement is simultaneously achievement-oriented, socially visible, and bodily expressive. In such settings, global SE may be relevant but insufficient: the remaining direct association is compatible with additional domain-specific evaluative processes. A stronger theory-building model would therefore treat the sociometer not as a single complete explanatory account but as one layer in a multi-level self-evaluative system, with global relational value and physical-domain appraisals potentially operating in parallel or sequence.

Third, the study brings an inclusion lens into models of exercise intention. Reputation safety, peer climate, stigma, and exclusion are not conventional variables in intention models, which more often emphasize attitudes, norms, perceived behavioral control, and self-regulation (Jha et al., 2024; Su et al., 2024). The present associations suggest that equitable willingness to exercise may also be embedded in whether students perceive the social environment as accepting enough for visible participation. Future PE-specific models can test whether psychological safety and mastery climate moderate the associations among peer devaluation, self-evaluation, and participation.

Accordingly, the theoretical contribution is best viewed as a qualified extension of sociometer theory rather than a confirmatory process test: the data are consistent with a sociometer-related statistical component, identify a sizable residual association that motivates boundary conditions, and connect social inclusion with motivation in visible physical contexts.

### 4.5 Potential practical implications for inclusive physical education

Because this study did not test any intervention, the following implications should be understood as practice-oriented hypotheses rather than demonstrated intervention effects. The findings nevertheless identify a coherent multilevel area for attention: reducing unnecessary negative peer evaluation while supporting self-worth and psychological safety across PE teaching, student support, and school policy.

For PE teachers, this means managing the social-evaluative conditions under which movement is performed. A mastery-oriented climate can reduce the salience of public ability ranking by emphasizing effort, learning, cooperation, and self-referenced progress (Ames, 1992; Braithwaite et al., 2011; Digelidis et al., 2003). Practical options include planned or rotating grouping rather than public captain selection, private or low-publicity corrective feedback, assessment focused on individual improvement, and multiple ways to demonstrate competence. A stigma-sensitive pedagogy further requires avoiding body-focused remarks, public labeling of low-skilled students, and unnecessary disclosure of sensitive peer difficulties; neutral competence-supportive language, confidential check-ins, and graduated entry into demanding tasks may preserve dignity without lowering expectations. Peer-climate management can reinforce these practices through explicit norms against ridicule, gossip, body commentary, and exclusion, together with cooperative goals and teacher-arranged groups that reduce repeated status-based marginalization.

Psychological safety also depends on coordinated support beyond the PE lesson. Class advisers, counselors, PE staff, and relevant student-support personnel can maintain confidential reporting and referral routes, respond proportionately to reputation-related conflict, and share only the information needed to protect the student. Counseling or mentoring may support coping and self-evaluation, while coordinated communication can help PE teachers adjust grouping, feedback, or participation demands without publicly identifying or stigmatizing the student. Such individual support should complement, rather than substitute for, efforts to improve the peer climate in which negative evaluation occurs.

At the school-policy level, codes of conduct can explicitly address relational aggression and reputation-based harassment, staff guidance can incorporate stigma-sensitive communication, and schools can review whether routine PE practices create avoidable public comparison or humiliation. Inclusive school-based movement opportunities can also be broadened through cooperative and small-group activities, active recess, non-competitive clubs, and other low-stakes options that provide meaningful choice without removing challenge. This multilevel emphasis is consistent with Ciaccioni et al. (2026), whose DE-PASS review indicates that adolescent physical activity research has concentrated largely on individual and interpersonal determinants, while highly integrated multi-domain approaches remain limited and intervention effects are generally modest or uncertain. The present findings therefore do not imply that changing self-esteem or peer norms alone will alter physical activity; future PE-specific interventions should test coordinated combinations of teacher practice, peer-climate management, student support, school policy, and movement-opportunity design while assessing both psychosocial outcomes and actual participation.

### 4.6 Strengths, limitations, and future directions

Several strengths of the present study should be acknowledged alongside its limitations. The analysis drew on a relatively large sample of 1,713 junior middle school students recruited from 10 schools in Sichuan and Chongqing, used established measures of NSG, SE, and EI, and evaluated the constructs with latent-variable structural equation modeling, reliability and validity checks, bootstrap estimation, and a demographic-covariate sensitivity analysis. Conceptually, the study extends adolescent physical activity research by examining a reputation-related interpersonal experience that is rarely included in conventional intention models and by connecting peer evaluation, self-worth, and school movement contexts. This focus is relevant to the holistic perspective emphasized by Ciaccioni et al. (2026), because it adds specificity to the interpersonal domain of modifiable determinants. At the same time, the present study should be viewed as one component of a broader socio-ecological account rather than as a comprehensive model of adolescent physical activity.

Several limitations are central to interpretation. First, all focal constructs were measured concurrently, so the proposed ordering NSG - > SE - > EI cannot be established. Cross-sectional indirect estimates can be biased when the underlying process is longitudinal (Maxwell and Cole, 2007; Maxwell et al., 2011). Plausible alternatives include reverse or reciprocal ordering: adolescents with lower EI or weaker engagement in visible school movement settings may experience more peer scrutiny; adolescents with chronically lower SE may perceive ambiguous social cues as more devaluing; and NSG, SE, and EI may be reciprocally associated over time. The common-method tests used here address systematic response covariance, not these temporal alternatives or unmeasured confounding. The indirect association should therefore be read only as a statistical decomposition of cross-sectional covariance.

Second, EI was general rather than PE-specific, and the study did not measure PE attendance, effort, enjoyment, engagement, playground activity, extracurricular school sport, sedentary behavior, or objectively assessed physical activity. The discussion of inclusive PE is therefore a contextual implication rather than a direct empirical finding about PE participation or adolescent physical activity behavior. This distinction is important in relation to recent determinant research such as Ciaccioni et al. (2026), which evaluates changes in physical activity and sedentary behavior across school, home, and mixed settings. Future studies should pair general EI with PE-specific intentions and behaviors, teacher-rated or observed participation, and objective activity indicators such as accelerometry. Such designs could test whether the present associations generalize to actual equitable participation and could address the well-documented intention-behavior gap (Feil et al., 2023; Rhodes and de Bruijn, 2013).

Third, the study relied on self-report data from the same respondents. Procedural remedies and statistical checks suggested that common method bias was unlikely to dominate the findings, yet same-source self-report designs cannot fully remove shared response tendencies, social desirability, mood-related reporting bias, or differences in sensitivity to ambiguous social cues. Future studies could combine adolescent self-reports with peer nominations, teacher reports, counselor records, or ecological momentary assessment. These additions would improve measurement triangulation but, by themselves, would still not substitute for longitudinal or experimental designs when temporal or causal questions are of interest (Podsakoff et al., 2003).

The measurement of NSG was brief and focused on generalized perceived frequency. This is an important boundary condition for interpreting the observed effect sizes. The measure does not distinguish gossip source, content, audience, or circulation scope and may therefore combine gossip that is directly relevant to exercise (e.g., body shape, athletic competence, or performance) with gossip that is less directly connected to physical activity (e.g., academic performance, personality, or unrelated peer relationships). Such content heterogeneity can attenuate a domain-specific association when only a subset of reported gossip is relevant to exercise contexts, while also making the substantive meaning of a one-unit increase in NSG less specific. Accordingly, the present coefficients should be interpreted as associations with generalized perceived gossip victimization, not with exercise-related gossip specifically. Future research should use more differentiated measures that distinguish classmates versus teachers, content domains, perceived audience, circulation breadth, and offline versus online transmission, and should directly assess exercise-relevant gossip. Such designs could also test whether physical self-concept, perceived physical competence, social physique anxiety, or fear of negative evaluation are more proximal correlates than global self-esteem.

The sample consisted of junior middle school students recruited by convenience sampling from 10 schools in Sichuan and Chongqing. This population is relevant to the research question, but the sampling frame was neither national nor probability-based. The findings should therefore be interpreted as pertaining to the sampled students and school contexts rather than generalized to all Chinese adolescents or all Chinese junior middle school students. School type, geographic setting, and school-specific sample sizes should be reported transparently because these characteristics help readers judge external validity. Cross-cultural and multi-region replication would be valuable because the meaning of gossip, reputation, self-esteem, PE climate, and exercise participation may vary across educational systems and peer cultures.

A related limitation concerns the nested sampling structure. Students from the same school, and potentially the same class, may share teachers, school policies, peer norms, PE environments, and broader school climate. Such intraclass dependence can reduce the effective amount of independent information and may affect standard errors and significance tests, especially for a small and marginally significant path such as NSG → EI. The final analysis should explicitly state whether clustering was modeled and, where cluster identifiers are available, report an appropriate cluster-adjusted or multilevel sensitivity analysis. Because only 10 schools were sampled, school-level variance estimates and cluster-robust inference would themselves have limited precision; future studies should recruit a larger number of schools and classes to support stronger multilevel inference.

An additional limitation is that the model is concentrated at the individual and interpersonal levels. The study did not assess environmental or organizational determinants such as facility access, scheduling, extracurricular provision, assessment policy, or school-wide movement policy. Ciaccioni et al. (2026) similarly observed that adolescent physical activity intervention research has measured individual and interpersonal determinants much more extensively than environmental components and has rarely achieved high integration across domains. The present findings therefore should not be interpreted as implying that peer climate or self-esteem is sufficient to explain adolescent physical activity. Future research should test cross-level models that combine psychological and peer variables with classroom, organizational, policy, and physical-environment factors.

Future research should also expand the model with exercise-specific self-evaluative constructs, multilevel determinants, and temporal designs. SE is broad, whereas PE and school sport may involve more proximal concerns about body appearance, motor competence, and peer observation. Multi-wave studies could compare global SE, social physique anxiety, physical self-concept, perceived physical competence, peer support, and psychological safety as parallel or sequential correlates over time. Experimental or quasi-experimental PE studies could test whether integrated packages combining mastery-oriented teaching, peer-climate management, confidential student support, and school-level policy improve psychological safety, PE participation, and actual physical activity, and whether any benefits differ for adolescents reporting reputational threat. Research on physical self-concept and social physique anxiety provides useful foundations for these extensions (Babic et al., 2014; Hart et al., 1989).

## 5 Conclusion

This study identified concurrent associations among perceived NSG, global SE, and general EI in Chinese adolescents. Higher NSG was associated with lower SE and weaker EI, and the statistically significant indirect component through SE represented 37.62% of the total standardized association. The larger direct component indicates that a sociometer-consistent SE account is meaningful but incomplete, leaving room for domain-specific self-evaluative and peer-climate processes. The findings are relevant to PE because school movement is visible, peer-observed, and inclusion-sensitive; however, PE participation was not measured directly. Accordingly, the results should be interpreted as correlational evidence that motivates longitudinal, experimental, and PE-specific research on gossip, stigma, psychological safety, and equitable participation rather than as evidence of causal effects. Future work should also determine whether coordinated teacher-, peer-, staff-, and policy-level approaches translate into psychologically safer participation and measurable changes in physical activity.

## Data Availability

The original contributions presented in the study are included in the article/Supplementary material, further inquiries can be directed to the corresponding author/s.
